# Three-Dimensional Computed Tomography for the Safe Management of Esophageal Foreign Body

**DOI:** 10.7759/cureus.87720

**Published:** 2025-07-11

**Authors:** Kazumasa Zensho, Tsuyoshi Shiina, Shohei Moriya, Tadashi Moriwake

**Affiliations:** 1 Pediatrics, Kurashiki Medical Center, Okayama, JPN; 2 Pediatrics, National Hospital Organization (NHO) Iwakuni Clinical Center, Iwakuni, JPN

**Keywords:** 3d-ct, computed tomography, endoscopic retrieval, esophageal foreign body, foreign body impaction, hard candy, pediatric emergency, procedural planning therapeutic approach, treatment safety

## Abstract

Foreign body ingestion is a common pediatric emergency, with esophageal impaction requiring prompt evaluation and management. This case presents a school-aged female patient who ingested a hard candy that became lodged in her esophagus. Despite the absence of respiratory distress, there was uncertainty about the exact location due to the radiolucent nature of the foreign body. Three-dimensional computed tomography (3D-CT) was performed after conventional radiographs proved non-diagnostic. The 3D-CT images clearly demonstrated the exact location of the candy in the upper esophagus, confirmed airway patency, and revealed its size (18×15×8 mm) and orientation. This detailed visualization guided the endoscopic extraction procedure. The foreign body was successfully removed without complications. This case highlights the value of 3D-CT in providing critical anatomical information for pediatric foreign body management, particularly when conventional imaging is inconclusive and precise localization is essential for safe intervention. While conventional radiography remains the first-line imaging modality, 3D-CT can provide valuable additional information in selected cases, particularly for radiolucent objects.

## Introduction

Foreign body ingestion represents a common pediatric emergency requiring prompt and accurate diagnosis followed by appropriate intervention [1,2]. In children, the most commonly ingested foreign bodies include coins, toys, batteries, and food items such as nuts and hard candy. Complications can range from mild discomfort to life-threatening conditions, including airway obstruction, esophageal perforation, and mediastinitis [1,3]. Distinguishing between foreign bodies in the airway and the esophagus is crucial, as the management differs significantly [2,4]. However, this differentiation can be challenging when clinical presentations exhibit substantial overlap, potentially leading to harmful interventions if misdiagnosed.

Conventional radiographs may prove inadequate for definitive diagnosis, particularly with radiolucent objects such as hard candy. Standard anteroposterior and lateral radiographs successfully identify only 50-75% of foreign bodies, with detection rates significantly lower for non-metallic objects [2,5]. Two-dimensional imaging has inherent limitations in localizing radiolucent foreign bodies. The limitations of two-dimensional imaging in accurately localizing foreign bodies and determining their relationship to surrounding structures have been well-documented [2]. The advancement of computed tomography (CT) technology, especially three-dimensional CT (3D-CT) reconstruction [5,6], now enables detailed visualization of the foreign body's precise location, its relationship to surrounding anatomical structures, and accurate assessment of its size and orientation [6,7].

We present a case where 3D-CT reconstruction proved instrumental in the evaluation and management of an esophageal foreign body when conventional imaging was inconclusive. This case demonstrates the clinical utility of 3D-CT in providing critical spatial information for diagnostic certainty and procedural planning, highlighting how advanced imaging with 3D-CT can potentially reduce unnecessary invasive interventions in challenging pediatric foreign body cases [8,9].

## Case presentation

A school-aged female patient presented to our emergency department with acute-onset severe laryngeal pain and an inability to swallow saliva following an accidental ingestion of hard candy. The patient was in obvious distress but exhibited no signs of respiratory compromise. Vital signs were within normal limits: heart rate 110 beats per minute, respiratory rate 24 breaths per minute, blood pressure 105/68 mmHg, temperature 36.8°C, and oxygen saturation 99% on room air.

Initial interventions, including back blows and the Heimlich maneuver, had been attempted prior to arrival but were unsuccessful in dislodging the suspected foreign body. Careful physical examination revealed normal bilateral breath sounds without stridor or wheezing. The oropharynx appeared normal with no visible foreign body. The patient could phonate but reported severe pain with any attempt to swallow.

**Figure 1:**
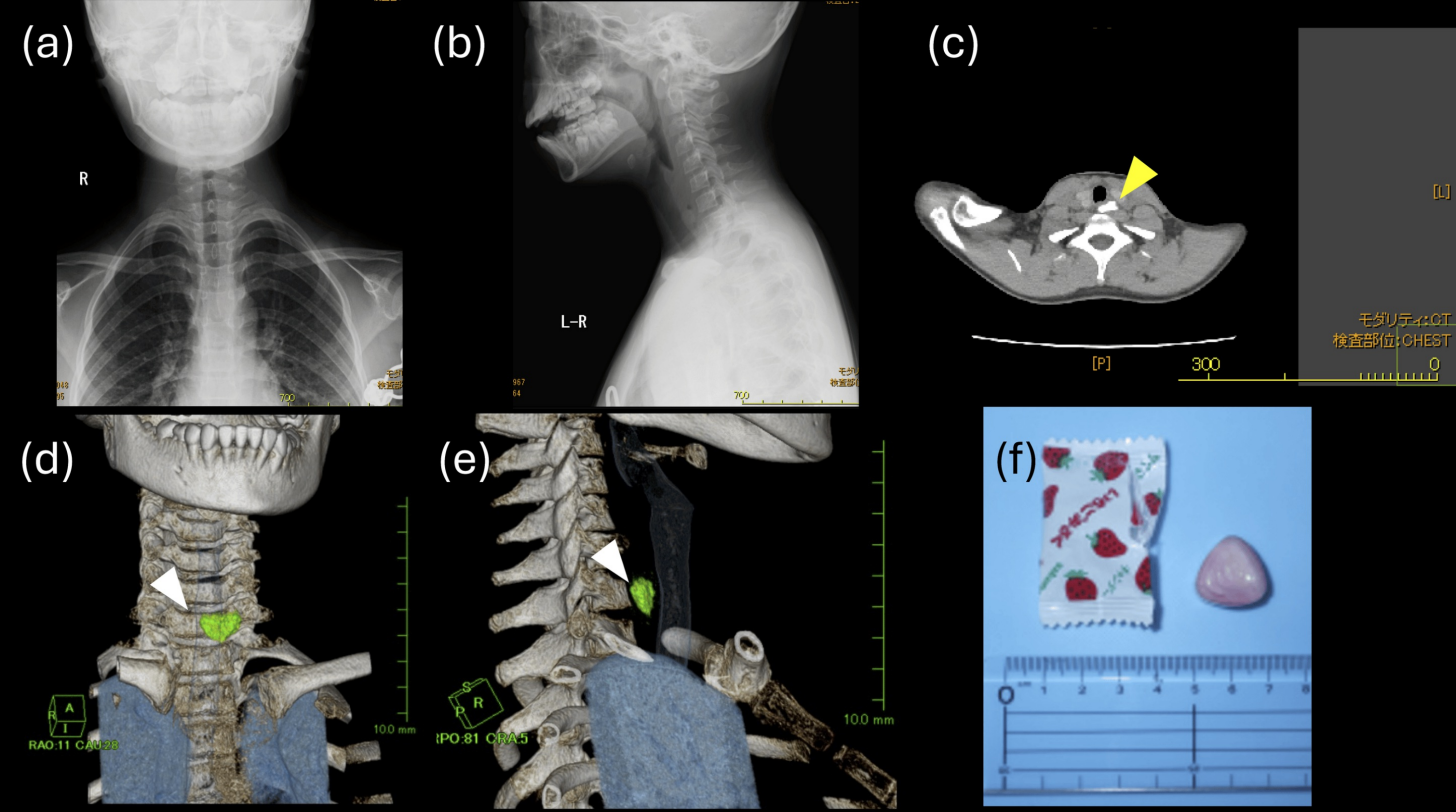
Imaging findings of the ingested foreign body (a) Frontal and (b) lateral radiographs showing no clear foreign body. (c) Axial CT showing the foreign body (yellow arrow) with a density similar to that of the vertebral bones. (d) 3D-CT reconstruction in frontal view and (e) lateral view showing the foreign body (green) lodged in the esophagus. The trachea is reconstructed in semi-transparent blue, confirming the absence of a foreign body in the airway. (f) The candy brought by the patient, matching the size of 18×15×8 mm and the shape of the foreign body visualized on the 3D-CT. CT: computed tomography; 3D-CT: three-dimensional computed tomography

Conventional radiographs of the neck and chest in frontal and lateral projections showed no evidence of a radiopaque foreign body (Figure 1a, 1b). Given the persistent symptoms and diagnostic uncertainty between esophageal and airway foreign bodies, a CT scan was performed. The axial CT images revealed a structure within the upper esophagus with density values of 300 Hounsfield units, consistent with those of the ingested candy (Figure 1c). 

3D-CT reconstruction was subsequently performed, providing three critical pieces of information: first, precise localization of the foreign body in the upper esophagus; second, confirmation of complete airway patency through transparent airway rendering; and third, accurate assessment of the foreign body's size (measuring 18×15×8 mm) and orientation (Figure 1d, 1e). The size and shape of the foreign body visualized on imaging matched the sample candy brought by the patient's family (Figure 1f).

After obtaining informed consent, urgent upper endoscopy was performed based on the 3D-CT findings. The endoscopist reported that the 3D visualization significantly facilitated planning the optimal approach angle and selection of appropriate retrieval instruments. The hard candy was successfully removed without complications. The patient was observed for several hours, demonstrated normal oral intake, and was discharged home with complete resolution of symptoms.

## Discussion

This case illustrates the diagnostic challenge presented by foreign body ingestion in pediatric patients [1-3,10], particularly when distinguishing between esophageal and airway involvement [2,3]. Foreign body ingestion accounts for significant pediatric emergency department visits annually, with esophageal impaction representing approximately 10-20% of cases requiring urgent intervention [1,3,10]. Despite normal respiratory parameters, the patient's inability to swallow and severe laryngeal pain created a diagnostic dilemma that conventional radiography could not resolve. The 3D-CT proved invaluable in establishing a definitive diagnosis and guiding appropriate management.

Several aspects of this case merit further discussion. First, the overlapping symptomatology between airway and esophageal foreign bodies highlights the importance of comprehensive evaluation [1,3]. While our patient maintained normal oxygen saturation and had no respiratory distress, the severity of symptoms necessitated ruling out potential airway involvement before proceeding with intervention [8,9]. Clinical presentations frequently overlap. However, as demonstrated in this case, symptom overlap between airway and esophageal foreign bodies can complicate clinical diagnosis, as documented in previous studies [2,3].

Second, the limitations of conventional radiography for radiolucent objects are well-documented across multiple studies. Hard candy, being primarily composed of sugar, does not appear distinctly on standard radiographs, a limitation that affects up to 25% of ingested foreign bodies [2,3,5]. When clinical suspicion remains high despite negative radiographs, additional imaging modalities should be considered according to established pediatric guidelines [8,9]. In our case, 3D-CT provided the anatomical information necessary for management [11,12]. This anatomical spatial information was instrumental in planning the endoscopic approach and selecting appropriate retrieval instruments according to established pediatric endoscopy guidelines, ultimately contributing to a successful outcome. The ability to visualize the foreign body's exact position relative to anatomical landmarks such as the cricopharyngeus muscle, aortic arch, and gastroesophageal junction enables precise procedural planning that is not achievable with conventional imaging.

The 3D-CT reconstruction provided the critical anatomical information described in our case presentation, enabling successful endoscopic retrieval [8,9,13]. This technology offers advantages over conventional imaging through multiplanar reformation and volume rendering techniques [11,12]. The anatomical precision afforded by 3D-CT reconstruction extends beyond simple localization.

While CT imaging involves radiation exposure, the benefit-risk assessment must be individualized according to established pediatric imaging principles [14]. Appropriate dose reduction protocols should be employed while maintaining diagnostic quality. The pediatric radiology community has emphasized the importance of optimizing CT protocols to minimize radiation dose while maintaining diagnostic quality [15]. In this case, accurate preoperative imaging prevented unnecessary bronchoscopy and reduced overall procedure time [10]. Studies have demonstrated that accurate preoperative imaging can reduce procedure time, decrease complication rates, and improve overall outcomes in pediatric foreign body management [10]. The gastroenterologist specifically noted that the 3D visualization facilitated planning of the optimal approach angle and selection of appropriate instruments for retrieval based on the precise anatomical relationships visualized.

Although not necessary in all cases of suspected foreign body ingestion, 3D-CT should be considered when conventional imaging is inconclusive and precise anatomical localization is critical for safe intervention [11,12]. The decision to perform CT should be made on a case-by-case basis, considering factors such as clinical presentation, the need for detailed anatomical information, the availability of alternative diagnostic modalities, the urgency of intervention, and adherence to radiation safety principles. Future research should focus on developing risk-stratified imaging protocols and evaluating the cost-effectiveness of 3D-CT versus empirical endoscopy in pediatric foreign body management [3,8-10].

## Conclusions

This case demonstrates the value of 3D-CT reconstruction in managing a challenging pediatric esophageal foreign body when conventional imaging was non-diagnostic. The technology enabled accurate diagnosis and guided successful endoscopic retrieval without complications. As a single case report, these findings cannot be generalized to all pediatric foreign body cases. However, this experience suggests that 3D-CT may be a valuable tool in selected cases where precise anatomical localization is essential for safe intervention, particularly with radiolucent objects. Future multicenter studies are needed to establish evidence-based protocols for the appropriate utilization of advanced imaging in pediatric foreign body management.
